# Supplementary material for: Prevalence and correlates of restless legs syndrome in men living with HIV
Source: PLoS One. 2021 Oct 1;16(10):e0258139. doi: 10.1371/journal.pone.0258139 (PMC8486089; doi:10.1371/journal.pone.0258139)
Supplement: Attachment — Submitted filename: ResponsetoReviewers_082921.docx [file pone.0258139.s001.docx]

09-04-2021

Dear Dr Ferri:

We thank you and the reviewers of our manuscript for the thoughtful feedback. Please find enclosed a point-by-point rebuttal to the reviewers’ comments.

**Reviewer 1**

*1. On line 82 page 4 authors should refer to the international diagnostic criteria for RLS (Allen RP, Picchietti DL, Garcia-Borreguero D, Ondo WG, Walters AS, Winkelman JW, Zucconi M, Ferri R, Trenkwalder C, Lee HB; International Restless Legs Syndrome Study Group. Restless legs syndrome / Willis-Ekbom disease diagnostic criteria: updated International Restless Legs Syndrome Study Group (IRLSSG) consensus criteria - history, rationale, description, and significance. Sleep Med. 2014 Aug; 15 (8): 860-73. Doi: 10.1016 / j.sleep.2014.03.025. Epub 2014 May 17. PMID: 25023924)*

**This reference has been added as suggested.**

*2. On line 85 page 4 authors should refer to the international diagnostic criteria for periodic movements of the lower limbs in sleep (Ferri R, Fulda S, Allen RP, Zucconi M, Bruni O, Chokroverty S, Ferini-Strambi L, Frauscher B, Garcia-Borreguero D, Hirshkowitz M, Högl B, Inoue Y, Jahangir A, Manconi M, Marcus CL, Picchietti DL, Plazzi G, Winkelman JW, Zak RS; International and European Restless Legs Syndrome Study Groups (IRLSSG and EURLSSG) . World Association of Sleep Medicine (WASM) 2016 standards for recording and scoring leg movements in polysomnograms developed by a joint task force from the International and the European Restless Legs Syndrome Study Groups (IRLSSG and EURLSSG). Sleep Med. 2016 Oct; 26: 86-95. Doi: 10.1016 / j.sleep.2016.10.010. Epub 2016 Nov 7. PMID: 27890390).*

**This reference has been added as suggested.**

*3. Line 146 page 6: it is advisable to specify the cutoff values of the ESS scale to be considered pathological.*

**This detail (ESS > 10) has been added in the Methods section as suggested. This metric has also been added to Table 4.**

*4. Line 155 page 7: the international diagnostic criteria for PLMS must be indicated in the bibliographic notes (already indicated in point 2), then replace in the bibliographic note 17.*

**This has been changed as suggested.**

*5. Line 255 page 12: the authors speak of "definite or indeterminate RLS", a denomination that however does not exist in the diagnostic criteria and in the definition of the pathology.*

**We have clarified that this RLS terminology is per the developer of the questionnaire and does not exist in the RLS diagnostic criteria. This has been explicated fully in the Methods and Limitations sections. However, we specifically retained this terminology because we solely utilized this questionnaire to determine the RLS categories. We have removed the language concerning RLS “diagnostic categories” to read RLS “categories”**

*6.* *Line 358 page 17: Add as another limitation of the study the fact that the authors did not also consider the severity of RLS, for example by means of the international Restless Legs Syndrome study group severity rating scale:
a. Walters AS, LeBrocq C, Dhar A, Hening W, Rosen R, Allen RP, Trenkwalder C. Validation of the International Restless Legs Syndrome Study Group rating scale for restless legs syndrome. The International Restless Legs Syndrome Study Group. Sleep Med. 2003 Mar;4(2):121-32.*

**We have added this to the Limitations subsection of the Discussion and added references as suggested.**

*7.* *Line 360 page 17: add that also the distribution of PLMS varies with age in patients with RLS (**Ferri R, DelRosso LM, Silvani A, Cosentino FII, Picchietti DL, Mogavero P, Manconi M, Bruni O. Peculiar lifespan changes of periodic leg movements during sleep in restless legs syndrome. J Sleep Res. 2020 Jun; 29 (3):* *e12896. doi: 10.1111 / jsr.12896. Epub 2019 Jul 16. PMID: 31313413) and therefore it would be interesting to evaluate this aspect in patients with HIV, also in the light of what is described in the discussion also on PLMS.*

**We have added this interesting point to the discussion and highlighted this potential future aim.**

**Reviewer 2**

*1.* *It could be interesting to know if RLS symptoms were chronic or intermittent adding informations on the frequency of RLS symptoms in patients with and without HIV.*

**One of the items of the CH-RLSq queries RLS symptom frequency over the last 12 months. We have added details about the response pattern to this item to the results section.**

*2.* *The authors found an interesting correlation between HIV disease duration and RLS. In these cases, has the patients been diagnosed before the study and asking for treatment?*

**There was no information collected during the MACS sleep ancillary study on whether individuals had a pre-existing diagnosis of RLS. The medication categories collected were broad (i.e., antidepressant, tranquilizers) making the adjustment for more RLS-specific (e.g., dopamine agonists) medications was not possible.**

*3. In the discussion session the authors reported increased RLS severity in patients with HIV in comparison with patients without HIV. What does it mean “severity” if any RLS severity scale has not been included in the study?*

**We have removed mention of “severity” of RLS throughout the manuscript. As has been correctly pointed out, the score of the CH-RLSq cannot be inferred to mean greater RLS severity. The lack of severity assessment of the CH-RLSq has been added as a major limitation of the instrument in the Limitations subsection of the Discussion.**

*4.* *Men with HIV and RLS had greater sleepiness, but once again the lack of excessive daytime time sleepiness is a supportive criteria for RLS diagnosis. The authors reported that sleep apnea was very common across all groups indicating that multiple sleep disorders may co-exist in people with HIV and that their impact may be cumulative. This aspect is interesting but it should be distinguished the impact of other sleep disorders from the impact of RLS itself.*

**The reviewer is correct in pointing out that most patient with RLS patients may not report excessive daytime sleepiness (reference). In fact, Table 4 now shows that 31% of men without HIV and RLS and 35% of men with HIV and RLS had pathologic levels of daytime sleepiness (ESS > 10). However, an important difference between our study and many studies exploring daytime sleepiness in RLS is that most have exclude individuals with comorbid sleep disorders (Kallweit U et al 2009; Fulda S et al 2007). Therefore, some of the sleepiness may originate from these other comorbid sleep issues or other comorbidities that may be associated with hypersomnia and RLS (i.e., depressive symptoms). We have highlighted this important difference in our sample in the Discussion.**

*5. Finally, I suggest to discuss some unexpected findings. For example, the authors reported a longer time in bed among patients with RLS. That is really strange because bed is usually the worst place for the onset of RLS symptoms.*

**This is a good suggestion for clarification. Many patients with RLS have comorbid insomnia and engage in behaviors that further impact their sleep adversely. By targeting these behavioral targets, a recent RCT showed the superiority of cognitive behavioral therapy for insomnia over sleep hygiene in improving sleep quality, efficiency, and insomnia symptoms in patients with RLS and comorbid insomnia (Song ML et al Sleep Med 2020 Oct;74:227-234). We have added this reference and point to the discussion.**

Once again, thank you for the gracious comments. We believe these changes have strengthened the manuscript considerably.

Sincerely,

Douglas Wallace and colleagues.
